# What Does It Mean?

**Published:** 1900-12

**Authors:** B. F. Arrington

**Affiliations:** Goldsboro, N. C.


### WHAT DOES IT MEAN?

BY DR. B. F. ARRINGTON, GOLDSBORO, N. C.

In the *Items of Interest*, August issue, 1900, under the heading "Snap-Shots at the National Association," the first paragraph reads as follows: "The National Association held its annual meeting at Old Point Comfort, the picturesque spot where the supposed amalgamation of the old American and Southern Associations occurred three years ago. The idea of having subdivisions of the Association, to be known as Eastern, Western, and Southern branches, it is well known now, was placed in the constitution as a means of inducing the Southern men to agree to union. From any other view-point the plan is bad, and as neither an Eastern nor a Western branch has ever been organized, it is to be hoped that in the not distant future the Southern men will themselves offer an amendment to the constitution abrogating the branches, in which event we will have a truly representative National Association."

In plain English, this is a frank confession of insincerity, trickery, and unfair dealing. In the interest of all concerned, I will ask, are we to understand by such expressions and open acknowledgment that fraud was premeditated and perpetrated under deceptive disguise to induce agreement to union? As the language quoted reads, such would seem to have been the design and action of some who were zealous in scheming and effort to affect arrangement for the organization of the National and union with the Southern as consummated at Old Point Comfort in 1897.

The expression, "it is well known now," was unfortunate, and reveals an ugly feature, for the presumption is clear and undeniable that if it is well known now, it certainly was well known then, during the planning and scheming for organization and union, at least to some of the active manipulators. It was nothing more nor less than a shameful act of deliberate deception and professional littleness, unworthy of the occasion and the work involved, and can but be censured and repudiated by all honest, fair-minded men in the profession.

A National Association, or any other professional organization, planned and established upon a basis of such trickery and unfair dealing can never succeed in development of grandeur for good.

As regards the expression, "From any other view-point the plan is bad," I will venture the opinion (strictly on a basis of equity) that from that view-point it has not and will not receive favorable consideration; on the contrary, it must be regarded far off the line of correct professional dealing.

That first paragraph of "Snap-Shots" must have been written hastily and rushed to the press-room without due consideration and reflection. In all charity, such we will hope was the case. But until explanation is made the language as recorded will admit of but one construction,—deception and fraudulent intent,—which is to be regretted. In the interest of the profession and for the credit of all concerned, as schemers in arranging for the organization of the National, we will hope there was in the hasty work on "Snap-Shots" some typographical errors that will admit of correction.

We can but hope and believe the spirit and intent of a large majority of the dentists who figured conspicuously for the organization of the National and union with the Southern at Old Point Comfort were honest in purpose, and not in any way participants in the perfidy indicated in the paragraph quoted.

The expressed hope that "in the not distant future the Southern men will themselves offer an amendment to the constitution abrogating the branches" is far fetched. No Southern man, a member of the Southern Dental Association, unless a hireling with a fixed price (there may be such, but I am unwilling to believe it), will ever offer such an amendment under existing circumstances.

To present this subject for consideration was the duty of some member of the Southern Dental Association who had been an active participant in advising and arranging for the union at Old Point Comfort; but as none (so far as I know) have ventured an expression, and by silence seemingly assent to and sanction the statements in "Snap-Shots," I therefore volunteer service, and feel justified in doing so as one of the original organizers of the Southern Dental Association. I must say, it is with sincere regret that I have felt it to be a duty to present and state the subject as above, but have done so from best motives and best wishes in behalf of the true interest of the profession, and for the sake of good feeling and harmony will express the hope that explanation may be made and no cause remain to justify belief that ungenerous and unfair dealing was purposely perpetrated in arranging for union of the Southern and National Associations.

In connection with the above it is legitimate to ask of knowing ones, Why the Eastern and Western Associations, corresponding to the Southern, as branches of the National, have not been organized? It was proclaimed, and distinctly expressed and understood, at the meeting at Old Point, before the organization of the National and union with the Southern was effected, that such organizations were to be established, and it was upon the faith of such understanding that the work of organization and union was successfully effected. This is a fact that no one can truthfully deny. It was even specified what territory each organization should embrace. So far there has been an evident want of good faith in not carrying out the arrangement and agreement as stipulated, and which was confirmed by a majority vote, all believing, or had the right to believe, that good faith and honest purpose was the basis of action. It is to be hoped that an equitable and satisfactory explanation can be offered, and that the National Association (purged and purified as needs may require) may develop into a grand organization for more advanced practical and scientific work and higher attainments in dentistry.

As a member of the Southern and National Associations, I have written the above in the general interest of the profession, hoping to stimulate sentiment and sanction for legitimate and fair dealing in all professional transactions.

